# Effects of water-deprivation stress on the expression of hepatic genes involved in blood pressure and stress response in chickens

**DOI:** 10.3389/fphys.2026.1919762

**Published:** 2026-09-11

**Authors:** Seong W. Kang, Chloe H. Sanchez, Rosie H. Whittle, Angela Perretti, Jaelen M. Cherry, Maricela A. Maqueda, Sara K. Orlowski-Workman, Young M. Kwon, Shawna L. Weimer

**Affiliations:** 1 Department of Poultry Science, Center of Excellence for Poultry Science, University of Arkansas, Fayetteville, AR, United States; 2 Department of Biological Sciences, University of Arkansas, Fayetteville, AR, United States

**Keywords:** blood pressure, broilers, gene expression, hypertension stress, liver, water deprivation

## Abstract

Water intake is crucial for livestock performance and welfare. Understanding the mechanisms underlying water homeostasis and stress responses during water shortages is essential for sustainable poultry production. This study examined how water deprivation (WD) affects hepatic genes related to blood pressure (BP) regulation and stress responses in broilers selected for high (HW) and low (LW) water efficiency. The genes analyzed included angiotensinogen (*AGT*), renin (*REN*), glucocorticoid receptor (*GR*), 11β-hydroxysteroid dehydrogenase-1 (*11β-HSD1*), and brain-derived neurotrophic factor (*BDNF*), measured in the liver. Birds from both lines underwent WD for 0, 12, and 24 h, with liver samples collected on days 38-40. The results showed that *AGT* and *REN* levels were higher in male than in female HW birds, indicating gender-specific expression of *AGT* and *REN* in the liver. The effects of 12 h and 24 h WD were more pronounced in HW than in LW birds, regardless of sex, implying a higher responsiveness of HW birds to WD stress. In both HW and LW, females exhibited higher *GR* and *11β-HSD1* levels than males. WD elevated *GR* and *11β-HSD1* expression in HW males but not in HW females, indicating a gender-specific stress response in the liver of HW birds. Conversely, LW females showed decreased *GR* levels after WD, and *11β-HSD1* was reduced after 24 h of WD in both sexes, suggesting a destructive stress response in LW birds. *BDNF* expression was three times higher in HW males than in HW females, with no significant gender difference in LW, suggesting a possible functional role for BDNF in the WD stress response and liver welfare. WD caused a time-dependent reduction in *BDNF* in both lines, indicating that water efficiency does not influence *BDNF* expression by gender. Overall, HW birds may demonstrate greater resilience in water homeostasis and BP regulation compared to LW birds. Hepatic *BDNF* expression levels in broilers may serve as a potential diagnostic marker of poultry health.

## 1 Introduction

Major livestock concerns will stem from water scarcity, so increasing production efficiency in domesticated animals and conserving water will be key to supporting population growth (Aloui et al., 2024; Popkin et al., 2010; El Sabry et al., 2023). Climate change is exacerbating water scarcity, which can be a critical limitation for the poultry industry (El Sabry et al., 2023). The FAO (Food and Agriculture Organization of the United Nations) has committed to addressing water scarcity in agriculture and promoting best practices for water use (https://openknowledge.fao.org/handle/20.500.14283/ca3945en). Furthermore, agriculture and poultry production are core components of livestock farming, and limited natural resources and global warming threaten these sectors (Tollefson, 2022).

Therefore, broilers were selected for divergent water efficiency: high (HW) and low (LW). This selection was based on the water conversion ratio (WCR), calculated as [WCR = water intake (g)/body weight gain (g)] (Lassiter et al., 2025). Broilers with the lowest WCR are considered more water-efficient and assigned to the HW line, while those with the highest WCR are assigned to the LW line (Aloui et al., 2024). Water deprivation (WD) reduces feed intake and growth performance in broilers and alters hen behavior in a gender-specific manner (Lassiter et al., 2025; Rault et al., 2016; Marks, 1987). During WD, a decrease in extracellular water volume increases plasma osmolality due to elevated solute concentrations, primarily sodium, and gender differences have been observed (Leib et al., 2016; Thornton, 2010; Stachenfeld et al., 2001). Inadequate management of dehydration and reduced blood volume may decrease organ perfusion, ultimately leading to organ failure (Kreimeier, 2000). The multisystem response to WD is coordinated and includes activation of the renin-angiotensin system (RAS). WD-induced BP regulation may be a sexually dimorphic trait (Connelly et al., 2022). However, it remains unclear how WD affects hepatic gene expression involved in BP regulation in broiler chickens and how this, in turn, contributes to hepatic function. WD is a significant stressor that leads to poultry mortality (Augustine, 1982; Baker-Cook et al., 2021). Stress plays a vital role in health and, depending on circumstances, can trigger physiological, cardiovascular, and neuroendocrine responses. The hypothalamic-pituitary-adrenal (HPA) axis has a substantial influence on water balance (Castle-Kirszbaum et al., 2023). Therefore, water balance is essential for the physiological stability of animals. The liver plays a vital role in maintaining electrolyte balance and cardiovascular function. The classical RAS is a complex proteolytic cascade involving enzymes, peptides, and receptors that regulate physiological functions, including BP and fluid balance. It also plays important roles in stress responses (Lubel et al., 2008). Recent studies in birds have shown that 24 h of WD increases plasma corticosterone levels (Scanes and Pierzchala-Koziec, 2024). In the liver, the GC receptor (GR) is essential not only for metabolic responses but also for general stress signaling, which may be vital for maintaining metabolic resilience and maximizing health benefits (Makris et al., 2025).

Brain-derived neurotrophic factor (BDNF) is a stress- and physical activity-dependent neurotrophic factor linked to emotional and cognitive functions and has been proposed as an indicator of animal health in domesticated animals (Rault et al., 2018). In mammals, higher BDNF concentrations are associated with greater stress resilience, and hippocampal BDNF enhances learning and memory. Additionally, BDNF concentrations in the brain and blood are correlated, offering the opportunity to use peripheral BDNF as a minimally invasive measure of enrichment effectiveness that reflects neural changes. Currently, little is known about whether hepatic BDNF affects the liver’s stress response to hypohydration in birds. Several studies suggest that hepatic BDNF may play a key role in maintaining liver innervation and hematopoietic functions (Genzer et al., 2017; Grzelak et al., 2023), and its levels can help evaluate liver function in humans with cirrhosis (Grzelak et al., 2023). These studies show that BDNF binding to hepatocytes activates catabolic pathways, such as fatty acid oxidation. Meanwhile, gluconeogenesis is inhibited, and glycogen storage is triggered. Studies in other species have demonstrated that low circulating BDNF levels are linked to metabolic disorders, including obesity, metabolic syndrome, and related conditions (Chaldakov et al., 2004; Motamedi et al., 2017).

In the present study, the hypothesis was that divergently selected HW and LW birds would differ in hepatic gene expression related to BP regulation and stress responses. Therefore, the results from these studies will provide insights into the effects of WD on BP regulation and stress responses in HW and LW broilers.

## 2 Materials and methods

### 2.1 Experimental animals, housing and design

The divergent genetic selection program for the HW and LW broilers was initiated with a base population of modern random-bred (MRB) Arkansas (Hiltz, 2021). The MRB population is maintained by random mating and represents a commercially available broiler from 2015. For divergent selection, 24 MRB sire families were used to assess water intake (WI), growth, and WCR (water conversion ratio, WI/BW) over 4 weeks using a low-flow water-monitoring system (Hiltz, 2021). Because WCR is a direct measure, selection was based solely on WCR, with sire families having the lowest WCR (designated as high water-efficient, HW) and the highest WCR (designated as low water-efficient, LW) reared separately.

Eggs from the 6th generation of HW and LW broilers were collected, incubated, and hatched at the University of Arkansas Poultry Hatchery. On hatch day (D), male and female chicks were individually wing-banded for line identification and weighed. Three main factors (line, gender, and WD) and their interactions (line x gender or gender x WD) were considered. On D0, chicks were randomly assigned by line at the University of Arkansas Poultry Research Farm’s Poultry Environmental Research Laboratory (total birds N = 120, with 10 birds per treatment, 12 treatments _HW/LW, male/female, 0 h, 12 h, 24 h WD). The study used a randomized complete block design with 4 replicate pens, each containing 15 birds. Chicks were kept at age-appropriate temperatures, starting at 33 °C at hatch (D0), decreasing to 25 °C by 14 d, and maintained at 25 °C through D49. The lighting program was 24 h light (L) and 0 h dark (D) (24L:0D) for D0 to D3, then 23L:1D for D4 to D7, and finally 18L:6D thereafter.

LW or HW chicks were placed into 24 floor pens (12 treatments, 12 pens per line, 15 birds/pen, 1.2 m x 2.4 m, total birds N = 360, stocking density 0.19 square meters per bird). Caretakers walked through each pen to ensure birds acclimated to human presence. Birds were raised on wood-shavings litter flooring with free access to feed and water during the treatments. They received a standard corn-soy starter diet and free-choice feed from local commercial breeding companies.

### 2.2 Liver tissue sampling before and after WD

In this study, we examined the effects of three WD treatments: 0 h, 12 h, and 24 h of WD on HW and LW broilers. Birds were restricted from water access in their pens as part of their treatment, while feed was provided as needed. On Ds 39 to 40, in each HW and LW line, four pens per treatment were subjected to 0 h, 12 h, or 24 h of WD. Five HW males, five HW females, five LW males, and five LW females were randomly sampled from each pen and humanely killed by cervical dislocation for liver collection. Sampling was conducted systematically to ensure that, at each WD duration (0 h, 12 h, and 24 h), HW and LW birds were collected simultaneously, minimizing potential effects of sampling time. Liver sampling was performed aseptically, promptly placed on dry ice, and stored to preserve cellular integrity until gene expression analysis.

### 2.3 RNA extraction and two-step real-time qPCR

Among the 10 birds in each treatment, sex distribution was uneven, with a minimum of 8 per treatment. Therefore, we used 8 birds per treatment for qRT-PCR to ensure an even number of samples. Total RNA was extracted from dissected frozen liver tissues of the birds (n = 8/treatment, 12 treatments _HW/LW, male/female, 0 h, 12 h, 24 h WD, total N = 96) using TRIzol^®^ reagent (Invitrogen Life Technologies, Palo Alto, CA, USA), followed by DNase I treatment and purification with the RNeasy mini kit (Qiagen, Valencia, CA, USA), as previously described. RNA quality and quantity were assessed by agarose gel electrophoresis and a NanoDrop 1000 (Thermo Scientific, Wilmington, DE, USA). Two micrograms of total RNA from liver tissue were reverse transcribed into cDNA using oligo(dT)16 primers and SuperScript IV reverse transcriptase (Invitrogen, Grand Island, NY, USA), as previously described (Kang et al., 2020). Specific oligonucleotide primers were designed with the PRIMER3 program (https://primer3.org) (accessed in October 2025). Primer sets for chicken angiotensinogen (*AGT*), renin (*REN*), *GR*, 11β-hydroxysteroid dehydrogenase-1 (*11β-HSD1*), and *BDNF* were designed, and conventional RT-PCR was performed to optimize annealing temperatures for each primer set 1). PCR products were analyzed by agarose gel electrophoresis (3%). Melting curve analysis and PCR efficiency for each primer set were validated using default settings on the ABI 7500 system (Applied Biosystems, Foster City, CA, USA). PCR efficiency was evaluated by calculating the efficiency, with 95–100% accepted for this study. A portion of the cDNA was used for quantitative real-time PCR (qRT-PCR) on an ABI 7500 system with Power SYBR Green PCR Master Mix (Invitrogen, Grand Island, NY, USA). Internal controls included chicken glyceraldehyde 3-phosphate dehydrogenase (*GAPDH*), *β-actin*, and *18S* ribosomal RNA (18S). Dissociation curves were generated at the end of amplification to ensure data quality. All qRT-PCR experiments were performed in triplicate, and the average cycle threshold (Ct) values were calculated. Delta-Ct scores for gene transcripts were normalized to *GAPDH*, *β-actin*, or *18S* and expressed as relative fold change using equation 2^−ΔΔCt (Livak and Schmittgen, 2001). The gene names, NCBI accession numbers, primer sequences, PCR product sizes, and annealing temperatures (Ta) used in this study are listed in **Table 1**.

**Table 1:** Primers used for RT-Qpcr.

Gene	GenBank #	Primer sequences (5’-3’)	Size (bp)	Ta(°C)
*GAPDH*	NM_204305	F: CTTTGGCATTGTGGAGGGTC R: CGCTGGGATGATGTTCTGG	128	58-60
*18S*	AF173612	F: CCCCTCCCGTTACTTGGAT R: GCGCTCGTCGGCATGTA	60	60
*β-actin*	L08165	F: CACAATGTACCCTGGCATTG R: ACATCTGCTGGAAGGTGGAC	158	54-60
*AGT*	NM_001396391.2	F: AAGAAGATTCGGTGCCCCAG R: TCCTCGGTGCTTGCGTTTAA	138	58
*REN*	XM_040652912	F: TTCGCCACCGCCCGCTGA R: AATCTCTCTCTCCCCAGCGA	80	58
*GR*	NM_001037826	F: GCCATCGTGAAAAGAGAAGG R: TTTCAACCACATCGTGCAT	95	54
*11β-HSD1*	XM_417988	F: CTGGGAACTGTCTGCACAAC R: GATTGCGAGGAACCATTTACAG	96	56
*BDNF*	NM_001031616	F: GACATGGCAGCTTGGCTTAC R: GTTTTCCTCACTGGGCTGGA	167	60

### 2.4 Statistical analysis

Statistical analyses were performed using JMP^®^ 14.0 (SAS Institute Inc., NC). Normality was assessed with the Shapiro-Wilk test. Data were analyzed with two-way ANOVA, using line (HW vs. LW) and gender (male vs. female) as classification variables, including their interaction. If the ANOVA revealed significant effects, means were compared with Tukey’s HSD multiple-comparison test to evaluate relative changes in gene expression among treatment groups for each gene. If the interaction was not significant, the main factors (line and gender; line and WD) were analyzed separately with Student’s t-tests. Data are presented as the mean ± SEM. A p-value of < 0.05 was considered statistically significant.

## 3 Results

### 3.1 AGT, REN, GR, and 11β-HSD1 gene expression in the liver of HW and LW birds

**Figure 1:**
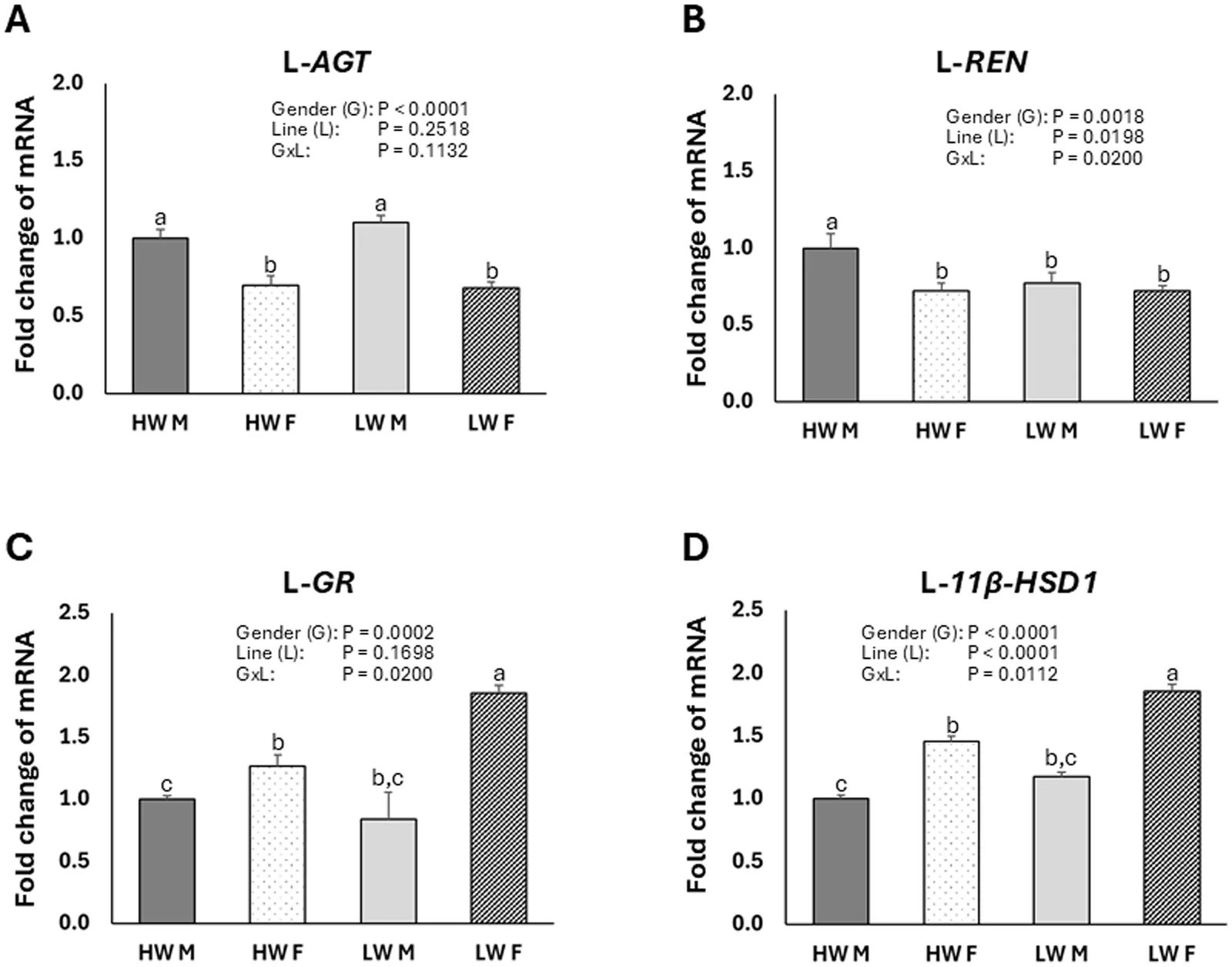
Relative mRNA expression of hepatic *AGT* (angiotensinogen), *REN* (renin), *GR* (glucocorticoid receptor), and *11β-HSD1* (11β-hydroxysteroid dehydrogenase-1) in male and female HW and LW broilers. Expression is shown for *AGT* **(A)**, *REN* **(B)**, *GR* **(C)**, and *11β-HSD1* **(D)**. Data are presented as relative fold changes using the ΔΔCt method, with *GAPDH* and *18S* as internal controls. Data were analyzed using two-way ANOVA and are presented as mean ± SEM (n = 8), with each gene set to 1.0 for HW M birds. The *p*-values for gender, line, and interactions are provided. Different lowercase letters indicate significant differences (P < 0.05).

There were no line differences in body weight, but significant gender differences were observed in both lines (data not shown). There was no significant gender x line interaction for *AGT* expression (**Figure 1A**). Significant gender x line interactions were observed for *REN*, *GR*, and *11β-HSD1* expression (**Figures 1B–D**). *AGT* expression in the liver was significantly higher in males of both selected lines compared to females (P < 0.05) (**Figure 1A**). Expression of *REN* in HW males was significantly higher than in the other groups of birds (P < 0.05) (**Figure 1B**). There were no significant differences in *REN* expression among HW females, LW females, and LW males. Expression of *GR* and *11β-HSD1* was significantly higher in both selected female birds compared to male birds (P < 0.05) (**Figures 1C, D**). *GR* expression in HW and LW males was 21% and 55% lower than in HW and LW females, respectively. Similarly, *11β-HSD1* expression in HW and LW males was 32% and 36% lower than in HW and LW females, respectively.

### 3.2 AGT and REN gene expression by WD in the liver of HW and LW birds

**Figure 2:**
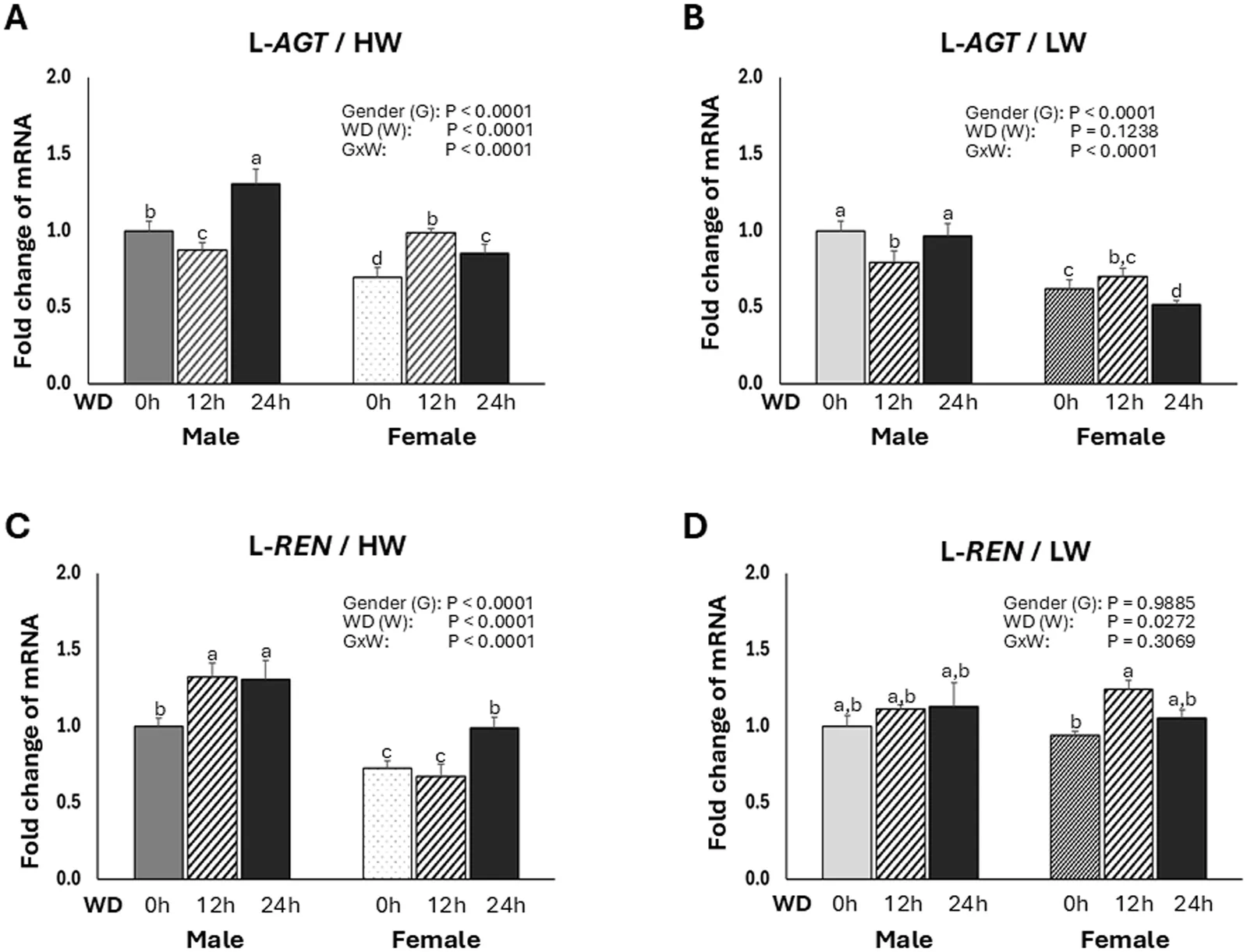
Relative mRNA expression of *AGT* (angiotensinogen) and *REN* (renin) after WD treatments in male and female HW and LW broilers. The mRNA expression is shown for *AGT*/HW **(A)**, *AGT*/LW **(B)**, *REN*/HW **(C)**, and *REN*/LW **(D)**. Data are presented as relative fold changes in expression levels using the ΔΔCt method, with *GAPDH* and *18S* as internal controls. Data were analyzed using two-way ANOVA and are presented as mean ± SEM (n = 8), with the value set to 1.0 for HW M 0 h birds for each gene. The p-values for gender, WD, and interactions are provided. Birds were subjected to 0, 12, or 24 h of WD as described in the Materials and Methods. Different lowercase letters indicate significant differences (P < 0.05).

The average body weight of WD birds was numerically lower at 12 h than at 0 h and significantly lower at 24 h than at 0 h in both genders and lines (data not shown, P < 0.05). Significant gender x WD interactions were observed for *AGT*/HW, *AGT*/LW, and *REN*/HW birds (**Figures 2A–C**). There was no significant gender x WD interaction for *REN*/LW birds (**Figure 2D**, P = 0.3069). WD-induced changes in *AGT* expression differed between the two selected lines (**Figure 2A**). In HW birds, males exhibited significantly higher *AGT* levels than females at 0 h (Gender P < 0.00001). WD duration increased *AGT* expression in HW birds. In HW males, *AGT* expression was significantly reduced at 12 h of WD compared to 0 h and peaked at 24 h of WD (**Figure 2A**). In HW females, *AGT* levels increased from baseline at 0 h to 12 h of WD (P < 0.05), but there was a significant decrease between 12 h and 24 h of WD. In LW birds, males showed a decrease at 12 h of WD and no difference between 0 h and 24 h of WD (**Figure 2B**). LW females showed a significant decrease in AGT expression at 24 h of WD compared to 0 h and 12 h of WD.

In HW males, WD increased *REN* expression at 12 h and 24 h relative to 0 h, but there was no significant difference between 12 h and 24 h. Across WD treatments, HW males consistently had higher *REN* expression than females (P < 0.05) (**Figure 2C**). In HW females, *REN* expression increased from 0 h to 24 h of WD, with 12 h levels approaching the 0 h baseline. LW males and females exhibited similar *REN* expression levels overall, but both were lower than those of HW males (**Figure 2D**). In LW female birds, *REN* expression increased at 12 h relative to baseline (P < 0.05), but levels at 12 h and 24 h did not differ. LW males showed a slight, non-significant increase in *REN* expression at 12 h and 24 h relative to 0 h (P > 0.05). Across all treatments, LW males and females showed similar statistical groupings in *REN* expression.

### 3.3 GR and 11β-HSD1 gene expression by WD in the liver of HW and LW birds

**Figure 3:**
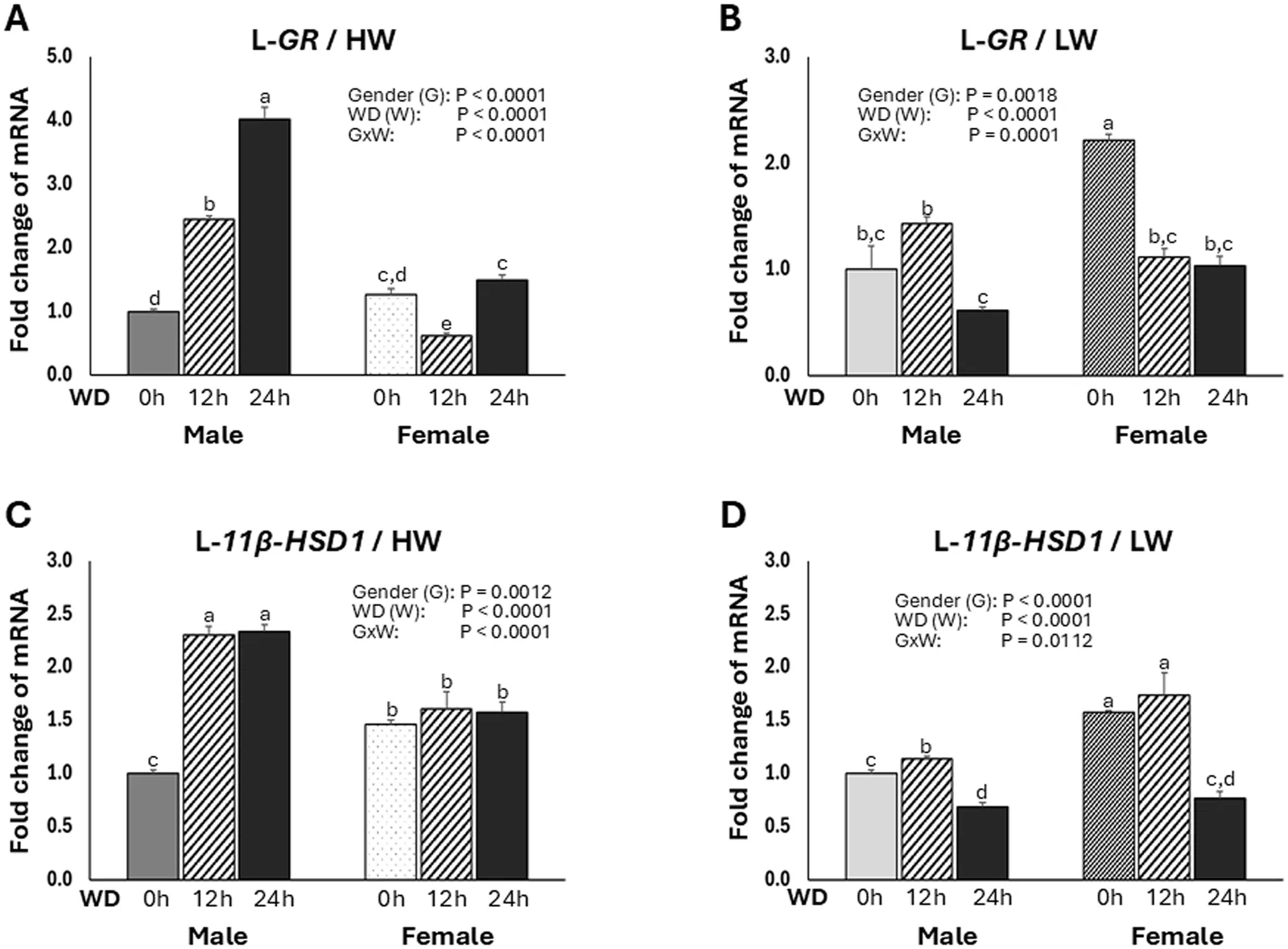
Relative mRNA expression of *GR* (glucocorticoid receptor) and *11β-HSD1* (11β-hydroxysteroid dehydrogenase-1) after WD treatments in male and female HW and LW broilers. The mRNA expression is shown for *GR*/HW **(A)**, *GR*/LW **(B)**, *11β-HSD1*/HW **(C)**, and *11β-HSD1*/LW **(D)**. Data are presented as relative fold changes in expression levels using the ΔΔCt method, with *GAPDH* and *18S* as internal controls. Data were analyzed using two-way ANOVA and are presented as mean ± SEM (n = 8), with HW M 0 h birds set to 1.0 for each gene. The P-values for gender, WD, and interactions are provided. Birds were subjected to 0, 12, or 24 h of WD as described in the Materials and methods. Different lowercase letters indicate significant differences (P < 0.05).

Significant gender x WD interactions were observed for *GR* and *11β-HSD1* expression in both HW and LW broilers (**Figure 3**). In HW birds, males had 20% lower *GR* expression than females. In HW males, 12 h and 24 h WD increased *GR* expression by 244% and 400%, respectively, compared with the 0 h baseline (**Figure 3A**). In HW females, *GR* expression decreased from 0 h to 12 h of WD (P < 0.05) and then increased significantly from 12 h to 24 h of WD. In LW birds, *GR* expression did not change at 12 h compared with 0 h WD (P > 0.05) and decreased significantly from 24 h to 12 h WD (**Figure 3B**). In LW females, WD decreased *GR* expression at 12 h and 24 h compared with 0 h, but there was no significant difference between 12 h and 24 h.

In HW male birds, *11β-HSD1* expression increased by 230% and 234% from the baseline at 0 h to 12 h and 24 h of WD, respectively (P < 0.05), but there were no significant differences in *11β-HSD1* expression in HW females across WD (**Figure 3C**). In LW birds, *11β-HSD1* expression was lowest at 24 h of WD compared with baseline 0 h and 12 h WD in both genders (**Figure 3D**).

### 3.4 BDNF gene expression by WD in the liver of HW and LW birds

**Figure 4:**
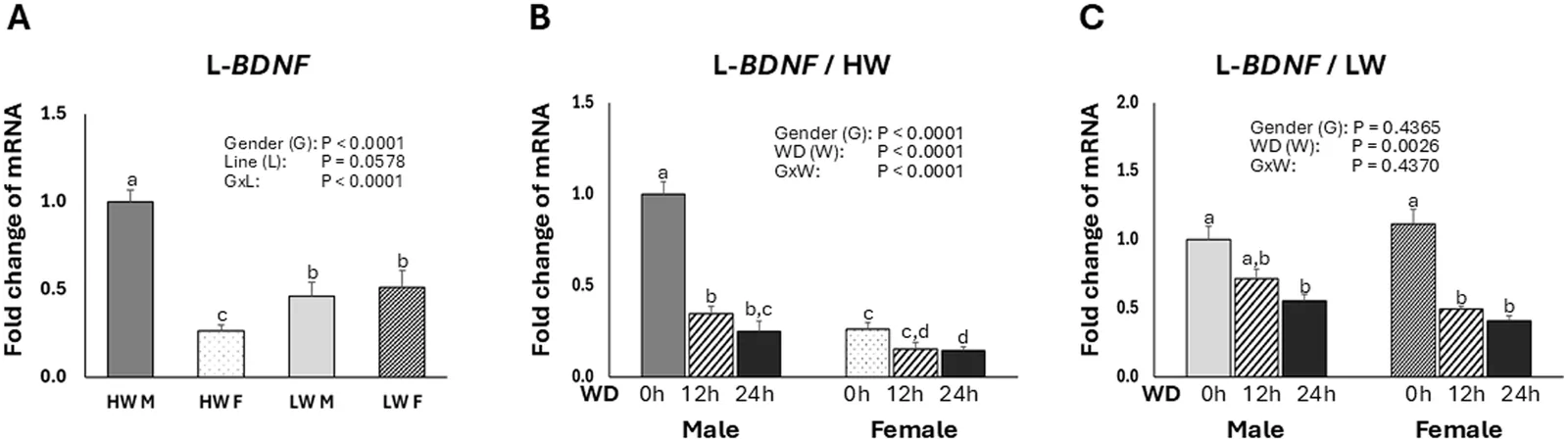
Relative mRNA expression of *BDNF* (brain-derived neurotrophic factor) in male and female HW and LW broilers before and after WD treatments. The mRNA expression is shown for *BDNF* **(A)**, *BDNF*/HW **(B)**, and *BDNF*/LW **(C)**. Data are presented as relative fold changes in expression levels using the ΔΔCt method, with *GAPDH* and *18S* as internal controls. Data were analyzed using two-way ANOVA and are presented as mean ± SEM (n = 8), with values set to 1.0 for HW M or HW M 0 h birds. The *p*-values for gender, WD, and interactions are provided. Birds were subjected to 0, 12, or 24 h of WD as described in the Materials and methods. Different lowercase letters indicate significant differences (P < 0.05).

A significant gender x line interaction was observed for *BDNF* expression (**Figure 4A**). A significant gender x WD interaction was observed in *BDNF*/HW broilers (**Figure 4B**, P < 0.0001) but not in *BDNF/*LW broilers (**Figure 4C**, P = 0.4370). Hepatic *BDNF* expression was 3.8-fold higher in HW males than in HW females (P < 0.05) (**Figure 4A**). However, there was no difference between LW females and males (P > 0.05). The duration of WD decreased *BDNF* expression in both genders of HW and LW birds. In HW female birds, there was a 45% decrease from baseline (0 h) to 24 h of WD (P < 0.05), but there were no significant differences between 12 h and 24 h of WD. In HW males, *BDNF* expression decreased by 66% and 75% at 12 h and 24 h of WD, respectively, compared with baseline (0 h), but there were no significant differences between 12 h and 24 h (**Figure 4B**). LW birds showed decreased *BDNF* expression from 0 h to 24 h of WD, with no significant differences between 12 h and 24 h (**Figure 4C**).

## 4 Discussion

Water scarcity can be a critical limitation on poultry production. Consequently, as broiler production grows, *ad libitum* water availability may not be guaranteed, and broiler chickens may be subject to water restriction for varying periods. One suggested solution to address water shortages is to develop water-efficient broilers, followed by confirmation of the effects of limited water access on production efficiency.

### 4.1 AGT, REN, GR, and 11β-HSD1 gene expression in the liver of HW and LW birds

The “classical” RAS is characterized by the breakdown of AGT, mainly produced by the liver, and the action of circulating renin (REN), which leads to the formation of angiotensin (Shu et al., 2021). REN is activated by decreases in BP, blood volume, or sodium levels and catalyzes the conversion of AGT into angiotensin I and II by angiotensin-converting enzyme. These components are activated to restore osmotic balance and blood volume. Overall, the RAS promotes sodium and water retention, thereby increasing blood volume and pressure (Correa et al., 2022). Although the classical RAS has been extensively studied and its essential role in regulating BP and fluid balance is well established, recent research has uncovered components of the alternative RAS (Garcia et al., 2023). Additionally, deficits in these components are hypothesized to significantly contribute to the development of hypertension, shifting some focus away from the classical RAS as the sole key player (Bader et al., 2024). In the present study, *AGT* expression was significantly higher in males in both HW and LW lines, whereas *REN* expression was higher in HW males than in HW females, indicating gender-specific patterns of *AGT* expression in both HW and LW broilers. The underlying mechanisms of BP regulation are believed to involve hypertensive disorders, interactions within the AGT-REN system, psychosocial gender interactions, and sex hormones (Connelly et al., 2022).

However, the understanding of these mechanisms remains limited. The present study aimed to investigate targeted, gender-specific gene expression involved in BP regulation in the liver. Gender differences in BP have been observed in other animals, and a previous avian study by Sturkie et al. reported that male White Leghorn chickens had considerably higher BP than females (Sturkie et al., 1953). Studies in other mammals have not demonstrated this, but Sturkie’s study suggested that differences in BP result from variation in hormone output and utilization (Sturkie et al., 1953). Stress alters GR expression in the liver of animals, including birds (Al-Mohaisen et al., 2000; Kang et al., 2017; Lattin and Romero, 2015). In mammals, GR and 11β-HSD1 regulate intracellular cortisol levels, emphasizing their fundamental roles in the pathogenesis of liver metabolic dysfunction (Ma et al., 2024). In avian species, *11β-HSD1* is expressed in a wide variety of tissues, including the liver, where it acts as a major regulator of GC (Katz et al., 2007; Klusonová et al., 2008). Regeneration of active GCs in cells via 11β-HSD1, rather than via circulating delivery, is critical for the development of the tissue-specific phenotype of GC excess (Kang et al., 2020). In the present study, we observed that selected female birds showed higher *GR* and *11β-HSD1* expression levels than males, indicating gender-specific differences in stress responses in both lines.

Higher GR and 11β-HSD1 expression levels are associated with stress susceptibility, reflecting heightened sensitivity of the HPA axis negative feedback loop (Bhatt et al., 2021). Therefore, in both HW and LW broilers, lower *GR* and *11β-HSD1* expression levels in the liver of males than in the liver of females may indicate greater stress resilience of males compared to females in the hepatic levels, as reported for gender-specific hepatic GR signaling in mammals (Duma et al., 2010; Quinn and Cidlowski, 2016).

### 4.2 AGT and REN gene expression by WD in the liver of HW and LW birds

To our knowledge, this study is the first to investigate how HW/LW lines and gender affect hepatic *AGT* and *REN* expression during restricted water intake. The findings demonstrate that 24 h WD alters hepatic *AGT* and *REN* levels, with acute WD-induced hypohydration increasing their expression in HW broilers. Such increases were not observed in LW broilers. Previous research has shown gender differences in water intake among broilers (Marks, 1987), suggesting that water consumption is regulated by gender-specific mechanisms. Similar responses in *REN* expression to 24 h WD were observed in HW and LW broilers, with 24 h WD-induced *REN* expression significantly increasing in both genders of HW broilers but not in LW broilers.

These results may suggest line-specific regulation of BP during WD. Notably, we observed that HW broilers showed significant changes in the 24 h WD-induced augmentation of *AGT* and *REN* expression in both genders, whereas LW males showed no significant increase, and LW females showed a significant decrease in *AGT* expression. In mammals, males showed a more sensitive arginine vasopressin response to changes in plasma osmolality than females under osmotic stimulation, suggesting that this greater sensitivity in males was associated with an increase in systolic BP (Stachenfeld et al., 2001), which appears to be consistent with our results showing upregulated intrinsic *AGT* and *REN* expression in males compared to females during WD. Thus, these results may suggest that HW broilers respond better to WD for BP control than LW broilers, thereby reducing the risk of further health complications.

### 4.3 GR and 11β-HSD1 gene expression by WD in the liver of HW and LW birds

The HPA axis influences it through corticotropin-releasing hormone (CRH) and glucocorticoids (GCs), which regulate renal water balance. It has been suggested that hypothalamic control of water balance arises from a complex interaction between GCs and water-regulatory mechanisms (Castle-Kirszbaum et al., 2023). GCs increase urine output and urinary sodium excretion and can lead to negative water balance (Castle-Kirszbaum et al., 2023; Baylis et al., 1990; Haack et al., 1977; Yoshida et al., 1988). Hypohydration induced by WD decreases plasma volume and increases plasma GC levels (Sawka et al., 2015; Zaplatosch and Adams, 2020). GCs have been reported to increase urine volume and urinary sodium excretion, leading to negative water balance and subsequent dehydration, which in turn induces hypoxia and oxidative stress responses in the livers of animals (Augustine, 1982; Baker-Cook et al., 2021; Castle-Kirszbaum et al., 2023; Baylis et al., 1990; Bal et al., 2022). In avian species, WD increases drinking behavior and plasma corticosterone (glucocorticoid) levels (Cain and Lien, 1985). Upregulation of hepatic *GR* in HW males at 12 h and 24 h of WD indicates a typical acute stress response. Chronic stress and continually elevated GCs reduce *GR* expression and sensitivity (Dickens et al., 2009; Jeffrey et al., 2012), suggesting that in the present study, the downregulation of *GRs* by 24 h WD in the liver of LW birds reflects a chronic stress response.

11β-HSDs play key roles in regulating GC action at the tissue level, with 11β-HSD type 1 (11β-HSD1) being the primary regulator of the tissue-specific effects of excess circulating GCs (Cooper and Stewart, 2009; Morgan et al., 2014). 11β-HSD1 converts inactive GCs into their active forms and has been linked to the development of metabolic syndrome. These enzymes are expressed in the liver of animals, including chickens (Harris et al., 2001; Kang et al., 2024). Tissue-based regeneration of GCs by 11β-HSD1, rather than circulating delivery, is critical in developing the GC-excess phenotype (Morgan et al., 2014). Increased *11β-HSD1* expression in the liver of HW males has been proposed as a possible mechanism for the significant amplification of GC levels. While the downregulation of *GR* was observed in the liver of LW birds, higher expression of *11β-HSD1* was seen in the liver of HW 24 h WD birds, indicating that active GC levels induced by *11β-HSD1* in the liver of HW males may be a key difference in stress responses between HW and LW lines.

### 4.4 BDNF gene down-regulation by WD in the liver of HW and LW birds

A positive correlation between BDNF levels in the brain and blood was reported (Rault et al., 2018). In fact, serum BDNF levels are associated with liver function in patients with cirrhosis (Shu et al., 2019). A large body of evidence suggests that GR actions largely overlap with those of BDNF, and that GR could regulate BDNF expression (Ichimura-Shimizu et al., 2024; Timmusk et al., 1993). The BDNF gene promoter in rodents contains several glucocorticoid response elements (Ichimura-Shimizu et al., 2024).

However, the molecular mechanisms underlying this regulation are not yet fully understood. BDNF expression in the brain and plasma of broiler chickens has been reported, and BDNF has been suggested to play a critical role and serve as a potential diagnostic marker of liver health (Ichimura-Shimizu et al., 2024; Kang et al., 2024; Timmusk et al., 1993; Bayraktar et al., 2020; Stawicka et al., 2021). Based on the results of the present study, it may be suggested that WD-induced GC and subsequent GR activation lead to downregulation of BDNF expression in the liver. Higher BDNF levels are ultimately associated with greater stress resilience, and BDNF in the brain enhances learning and memory (Batallas et al., 2025). A recent study showed a significant role for BDNF in the relationship between pro-inflammatory cytokines (IL-6, IL-15, IL-18) and declarative memory performance, in a gender-dependent manner (Batallas et al., 2025; Chan and Ye, 2017). Interestingly, sex-specific differences in hepatic *BDNF* expression were observed between HW and LW males and females in the present study, with higher expression in male HW than in male LW, and in female LW than in female HW. Stress-induced BDNF expression shows significant sex-dependent variations in mammals (Chan and Ye, 2017).

The results of the present study show downregulation of BDNF expression at 12 h and 24 h of WD, which indicates that BDNF may serve as a marker of the WD response in maintaining liver function and modulating stress responses in both HW and LW birds, as higher BDNF levels are positively correlated with greater stress resilience (Batallas et al., 2025; Ichimura-Shimizu et al., 2024).

### 4.5 Limitations of the present study

The present analysis was restricted to genes associated with BP regulation and stress responses; therefore, the findings should be interpreted with appropriate caution. Moreover, gene expression data do not necessarily correspond directly to protein abundance or biological activity. Physiological parameters, including plasma corticosterone, BP, and osmolality, were not evaluated, limiting the extent to which gene-expression changes can be directly linked to functional outcomes. Furthermore, the analysis was confined to hepatic tissue, although water homeostasis and stress responses involve multiple organs, including the kidney and central nervous system, which should be considered in future investigations.”

## 5 Conclusion

**Figure 5:**
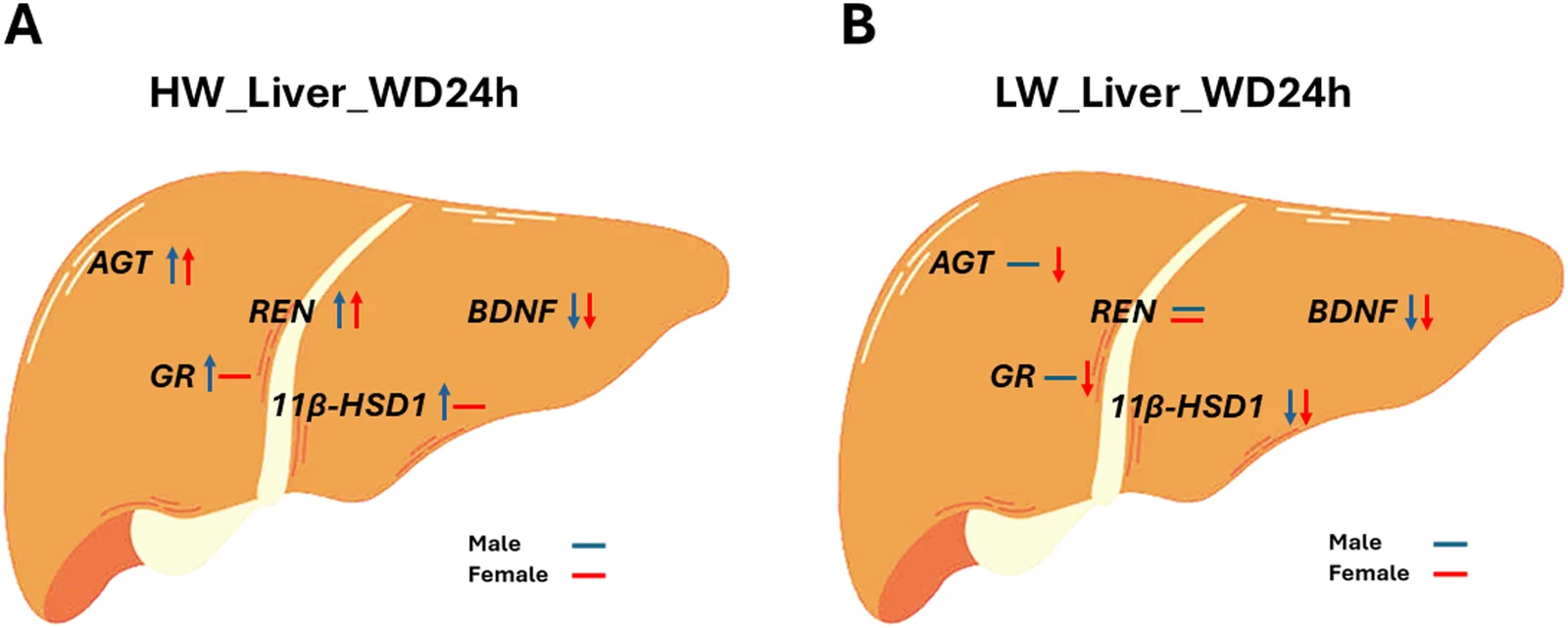
Schematic overview of effects of 24 h water-deprivation on the hepatic gene expression involved in the blood pressure and stress response compared to 0 h controls of HW_Liver_WD24h birds **(A)** and LW_Liver_WD24h birds **(B)**. The red arrow/line indicates female; the blue arrow/line indicates male. The up arrow means up-regulation of gene expression; the down arrow means down-regulation of gene expression. The side-to-side arrow indicates no change in gene expression. The following abbreviations are used in the figure: HW, high-water efficient broiler chickens; LW, low-water efficient broiler chickens; WD24h, water-deprivation 24 hours; *AGT*, angiotensinogen; *REN*, renin; *GR*, glucocorticoid receptor; *11β-HSD1*, 11β-hydroxysteroid dehydrogenase-1; *BDNF*, brain-derived neurotrophic factor.

This study examined how WD regulates genes involved in BP and stress responses in the livers of broiler chickens selected for high (HW) and low (LW) water efficiency (**Figure 5**). Correlated expression of *AGT* and *REN* in the liver may indicate a physiological mechanism for controlling BP during dehydration via extrarenal organs. Maintaining *GR* and *11β-HSD1* responses in HW males may be important for stress management. Notably, water-efficiency selection did not affect *BDNF* expression, and the WD-induced decrease in *BDNF* may provide evidence for a critical role for BDNF in this condition. Overall, WD appears to stimulate hepatic RAS components and stress-response genes in HW birds. Continued research could deepen our understanding of water balance in broilers and aid in developing strategies to enhance resilience to water shortages, such as genetic selection of water shortage-resilient broilers based on *BDNF* as a WD response marker.

## Data Availability

The original contributions presented in the study are included in the article; further inquiries can be directed to the corresponding author.
